# Supplementary figures and images for: The Aggregated Gut Viral Catalogue (AVrC): A unified resource for exploring the viral diversity of the human gut
Source: PLoS Comput Biol. 2025 May 2;21(5):e1012268. doi: 10.1371/journal.pcbi.1012268 (PMC12068713; doi:10.1371/journal.pcbi.1012268)

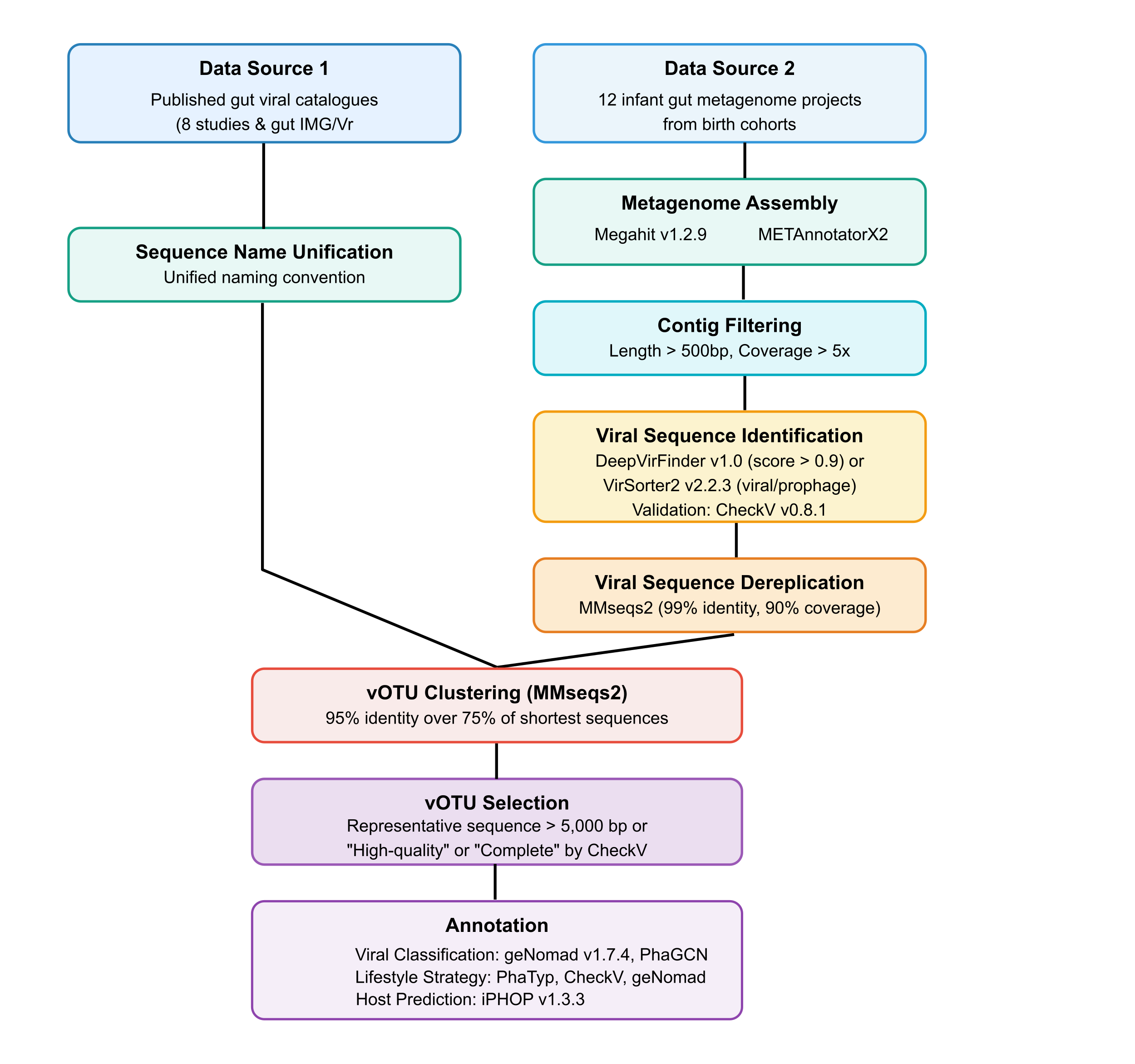

Supplement: S1 Fig — (TIFF) [file pcbi.1012268.s003.tiff]
