# Supplementary material for: The Aggregated Gut Viral Catalogue (AVrC): A unified resource for exploring the viral diversity of the human gut
Source: PLoS Comput Biol. 2025 May 2;21(5):e1012268. doi: 10.1371/journal.pcbi.1012268 (PMC12068713; doi:10.1371/journal.pcbi.1012268)
Supplement: Attachment — Submitted filename: Responses to reviewers.docx [file pcbi.1012268.s006.docx]

Reviewer #1: This article, a valuable tool for analyzing human gut microbiome viromes, is proof of the authors' remarkable effort. We highly appreciate their work in classifying, validating, and annotating the sequences obtained for the human gastrointestinal tract virome.

However, to be accepted, it needs to review a series of comments in the attached file.

I will be honored to review the revised version of this manuscript to proceed with its possible publication in the journal PLOS Computational Biology.

*We thank the reviewer for their time and helpful comments. We think the reviewer’s comment helped us clarify the text and improve on the readability of our manuscript. Below are the point by point answers for each of the reviewer’s comments:*

**Short abstract:**

- Can the authors add the link to this database?

*Thank you, now the link to the resource is added in the abstract and author summary*

**Abstract:**

- L26: The conditions of infants' intestinal microbiomes vary depending on diet and lifestyle. Could you add information about which country the data is from? If there is a lot of data, it could be from a certain region or continent.

*Thank you, we have now added information about the source of these infant samples in the abstract (L25-27). Given the abstract length limitation, we limited the information to give an idea of the size of the dataset and origin without too many details.*

- L46: Please add the link for this tool

*Thank you for pointing this omission. Now the link of the resource has been added in both the abstract and the author summary.*

**Introduction:**

- L83: In the authors' experience, what is the goal of directing research efforts to investigate the infant virome?

*Thank you for this important question. We believe that the infant virome is particularly relevant given its potential role in early-life microbiome development. This knowledge gap is significant because the infant gut represents a critical window for microbiome establishment, with long-term consequences for health. Previous studies have suggested that bacteriophages can shape the early colonization and succession patterns of bacterial communities and have a potential role in the infant’s immune system development. Given that most large-scale viral mining efforts have focused on adult populations, our inclusion of infant samples from 12 studies across 9 countries substantially expands the representation of this crucial developmental period in viral cataloguing efforts, helping to address an important research gap in the field.*

*We now added a sentence to clarify this in the introduction L85-87: “Despite the viral diversity of infant associated viromes (18) and the potential biological importance of the viral community in the infant gut microbiota acquisition and maturation (19,20), infant associated viruses have been largely under-explored in large-scale viral mining efforts.”*

- L86: Like a benchmark? Please revise this article. <https://link.springer.com/article/10.1186/s13059-024-03236-4>I suggest that the authors develop this study in the form of a "benchmark" of several tools for the detection of human gut virus

*Thank you for pointing this very interesting paper out. We agree that benchmarks of the computational tools used for viral sequence detection have been recently published, including the paper mentioned by the reviewer, and our own benchmark effort (*[*10.3389/fmicb.2023.1078760*](https://doi.org/10.3389/fmicb.2023.1078760)*). However, if the methods for viral sequence detection in metagenomes have been benchmarked, the resulting databases and catalogues that each leveraged different tools and approaches for viral mining have to date not been comprehensively compared. We clarified this point in the introduction L88-92 and added the references to these relevant benchmarks: “Large-scale viral mining efforts in human gut microbiome have been enabled by the development of viral mining computation tools and algorithms (3,6,8,21), each with their own strength and limitation (22,23). Despite the value of large-scale viral mining efforts in the human gut virome, there is currently no consistent comparison of the quality, diversity, and overlap of the human gut viral catalogues”*

**Results:**

**SECTION1:**

- L157: Which ones? please add these data as supporting information.

*Thank you for this suggestion. We now added a supplemental file 2 that details the information about these high prevalence vOTUs and mentioned L152.*

**SECTION2:**

- L161: This information can be useful in the abstract. Please consider adding it

*We agree with the reviewer and was added this information in the abstract*

**SECTION3:**

- L185: What about the 49% that must be added to these numbers to get the 100%? please clarify

*Thank you for raising this omission. We clarified that the rest of the sequences are assessed as low quality or could not be evaluated by CheckV L196-200: “The first release of the AVrC contains a total of 1,018,941 unique sequences clustered into 449,859 vOTUs, with 8% complete (n = 36,802), 21% high (n = 93,290) and 22% medium (n = 98,374) quality representative sequences, the rest of the sequences classified by CheckV as Low quality or could not be assessed by the tool (****Fig. 3A****)”*

- L188: This percent refers to 449859 vOTUs? please clarify

*Yes, absolutely. This is now clarified. L200: “Importantly, most vOTUs of the AVrC are singleton (65% of the 449,859 vOTUs, n = 294,300)”*

- Fig3 legend: In this panel, authors must declare the total number of catalogs used to prepare the AVRC database

Thank you for this comment. The legend was indeed incomplete. Now the number of catalogues and additional samples are detailed in the figure 3 legend. The legend now reads: *The AVrC included 9 previously published catalogues and resources (10,11,17,16,12,18,13,14,15) and more than 7,000 additional infant gut metagenomes (PRJEB70237, PRJNA345144, PRJEB32135, PRJEB6456, PRJNA384716, PRJNA473126, PRJNA290380, PRJEB42363, PRJNA695570, PRJEB32631, PRJNA497734, PRJNA489090).*

- L205: What is the logic behind these percentages, in no way does the sum add up to 100% or the 68% mentioned at the beginning of the sentence.

*Thank you for pointing this, the original sentence was indeed confusing, and some counts were wrong (some vOTUs were counted twice when iPhop predicted several potential host species). We clarified these counts as followed L227-232: “Using iPhop, 305,179 (68% of the total 449,859 AVrC vOTUs) of the vOTUs could be associated to at least one predicted prokaryotic host, and the predicted hosts for these viral species were part of the Bacillota (40% of the total 449,859 AVrC vOTUs n = 179,535), Bacteroidota (14%, n = 61,043); Pseudomonadota (5%, n = 23,173) and Actinobacteriota (4%; n = 16,795) phylum, corresponding to the major bacterial taxa found in the human gut (****Fig. 3C****)”*

- L227: The information deposited in the GitHub repository is correct. However, there are areas of opportunity in that it does not have a tutorial containing a set of test data, which allows the user to use the tool and obtain results that can be replicated. Another issue that should be taken into account is that the tool needs to have adequate documentation. If you have doubts about the documentation, please review the well-structured and comprehensive documentation of the programs developed in R Bioconductor. This resource can provide valuable insights and guidance for improving our own documentation. <https://contributions.bioconductor.org/docs.html>

*Thank you for these very good suggestions. We agree with the reviewer that the documentation was too light and would not allow for a full reuse of the resource. Considering this comment and the second reviewer’s comment we decided to re-organize the way we make the AVrC accessible.*

*- The database is directly accessible in Zenodo, along with a description of its content and associated data*

*- To help with the accessibility of the AVrC we developed a CLI toolkit that allows users to download and subset the dataset. This replaces the Shiny App we had developed before. Indeed, as the reviewer 2 noted, the app was slow and limited by the web implementation. The functionalities are maintained through the CLI but are now more reactive and able to scale up to the dataset size. The CLI toolkit is available at* [*https://github.com/aponsero/AVrC_toolkit*](https://github.com/aponsero/AVrC_toolkit)

*- The documentation for both the database and the CLI along with a tutorial on how to use the CLI have now been added in the Wiki section of the Github repository (*[*https://github.com/aponsero/AVrC_toolkit/wiki*](https://github.com/aponsero/AVrC_toolkit/wiki)*).*

**Discussion;**

- L252: So, this part must be made clear when presenting the tool because, at first, its use can be poor when it comes to microbiome samples from populations that are not represented within the database.

*We agree with the reviewer, and clarified that this was our rationale in our mining effort of infant microbiome samples*

*L94-99: “Here, we aimed to (1) survey previous viral mining efforts in human fecal metagenomes, assess their quality, diversity and overlap; to (2) leverage several large scale infant cohorts to explore the viral diversity associated to infant gut microbiome; and to (3) harmonize and aggregate all currently available gut viral catalogues in a unified resource that users can leverage to compare new putative viral sequences to previous mining efforts.”*

*We also clarified that the current limitation of the catalogues and mining efforts in the limitation and conclusion paragraph: L339-345: “The overview of previously published mining efforts highlighted several gaps and biases in our current understanding of the human gut virome. First, the geographical bias towards adult western populations can be addressed by actively incorporating mining from underrepresented age groups and regions. While in this first version of the AVrC we have expanded coverage by including infant samples from 9 different countries, the viral diversity from certain geographic regions and demographic groups remains underrepresented, and results should be interpreted accordingly when analyzing samples from these populations.”*

- L269: What do the authors suggest could be used to capture the most incredible diversity of viruses in the human gut?

*Thank you for this important question. We believe that capturing the full diversity of human gut viruses will require a multi-faceted approach:*

1. *Harmonizing existing resources through efforts like the AVrC to maximize the utility of already-discovered viral sequences*
2. *Expanding sampling to underrepresented populations and age groups*
3. *Employing complementary methodological approaches including the combined use of viral enrichment techniques and bulk metagenomics and the integration of long-read sequencing technologies alongside short-read approaches to allow for the recovery of more complete viral genomes*

*We have added this perspective to the discussion section: L306-309 '’Future efforts to capture the full diversity of human gut viruses should combine the harmonization of large-scale gut viral catalogues, the exploration of underrepresented populations, the integration of long-read sequencing technologies, and should be able to adapt to the novel developments in both computational and experimental approaches"*

- L283: In this last sentence, I recommend that the authors approach the International Committee on Taxonomy of Viruses: ICTV to be able to feed their tool and join forces in the interest of classifying and annotating human intestinal viruses in a better way. <https://ictv.global/>

*Thank you for this excellent suggestion. We agree that engaging with the ICTV would be valuable for improving viral classification and annotation. Our immediate plans include finding synergies between the AVrC and the INPHARED database (10.1089/phage.2021.0007), which implements ICTV taxonomy for bacteriophages. While the two resources have distinct purposes - INPHARED focuses on high-quality cultured phage genome sequences and AVrC aggregates metagenome-derived viral sequences - establishing connections between them would support standardized taxonomic annotation.*

*We have added a reference to this potential collaboration in the discussion L320-321: “Comparisons of the AVrC with culture-based viral genomic resources such as the Inphared database (33) would allow to explore the diversity of phages currently not captured by viral culture approaches.”*

**Methods:**

- L301: this refers to github repository?, please clarify

*Thank you for pointing this out. Now a link to the toolkit github repository was added.*

**Supplemental**

- In the supporting excel file, the VLP column of the first sheet must be "False" instead of "Falso"

Thank you for pointing this issue out. Now the supplementary file is corrected.

Reviewer #2: Dear authors,

The manuscript titled "The Aggregated Gut Viral Catalogue (AVrC): A Unified Resource for Exploring the Viral Diversity of the Human Gut" presents a significant effort to unify and enhance resources for studying the gut virome. The authors have systematically analyzed existing viral sequence catalogues and integrated new datasets to create a more comprehensive database for studying the virome in the human gut. The study is well-structured, and presents novel insights. There are areas where the manuscript could be improved for clarity, methodological rigor, and presentation. Please find below some comments to be addressed.

*We thank the reviewer for their time and helpful comments. We think the reviewer’s comment helped us improve on both the readability and the figures of our manuscript. We additionally considered the reviewer points on the limitations of the Shiny App and we decided to develop a CLI toolkit that would perform the same functionalities than the Shiny App but in a more scalable manner. Below are the point-by-point answers for each of the reviewer’s comments:*

**Introduction:**
- Line 86: Quality assessments and diversity analyses were presented throughout the manuscript but there weren't any measures on completeness explicitly. Is your measure of completeness based solely on overlaps between databases? Consider clarifying or revising this statement.

*Thank you for this comment, we agree with the reviewer that the term was not representative to the analysis performed. We now corrected “completeness” by “overlap” L91.*

**Results**

**SECTION1:**
- Lines 107-111: Briefly describe your method for estimating the number of fecal samples, countries, sample screening, health status, and overlaps in catalogues.

*Thank you for this suggestion, we now added a brief description of the method used. L113-116: “We retrieved the list of sample and sequences included in each catalogue from their respective supplemental material and manually curated metadata information about sample country of origin, health status associated with each individual sample from NCBI and ENA, with full details provided in Supplemental File 1”*

- Line 148: Specify which catalogue contains 82% of the unique sequences.

*Thank you for this comment. The sentence was unclear as we meant here that 82% of the vOTU could not be detected in more than one catalogue. We clarified this sentence to L139; “Strikingly, 82% of vOTUs (n = 195,153), were present in only one of the catalogues...”*

- Lines 149-151: The explanation regarding the relationship between resource size and shared sequences is unclear. MGV is not the largest catalogue. Shouldn’t IMG/VR and GPD, being larger, have higher shared sequences? Based on Fig. 1C, IMG/VR and KGP show higher shared sequences, while GPD (142K sequences) shows fewer. Clarify this reasoning.

*Thank you for pointing this out. Indeed the paragraph was really confusing as the text referred to the absolute number of vOTUs but the figure showed the proportion of vOTUs shared between catalogues, masking the large difference in size of the different resources. While the total IMG/VR is indeed larger than all the other catalogue, we only selected vOTUs that were assembled from Human gut in this resource, making MGV the largest resource integrated into the AVrC.*

*To clarify this section and to help the consistency between text and figures, the section now reads: L141-144: “The MGV and GPD were found to have the largest overlap in absolute number of sequences (n=15,453), which can be explained by the large size of the two resources, and the overlap in the samples included in these two mining efforts (Fig 1B).”*

**SECTION2:**
- Line 167: Figure 2 only has one panel; remove "A"

*Thank you, now removed*

**SECTION3:**
- Line 182: Justify the inclusion criterion for vOTUs (>5000bp). Can shorter sequences still provide valuable information? For example, 16S v3/v4 OTUs are ~1000bps but can still be used with high confidence.

*Thank you for raising these important points. Although shorter sequences could provide valuable information, several benchmarks showed that the correct classification of short contigs is still challenging [see* [*https://doi.org/10.3389/fmicb.2023.1078760].*](https://doi.org/10.3389/fmicb.2023.1078760%5d.) *To limit the possible false positive detection, we decided to have a minimum length of 5kb. We clarified this in the main text: L193-194: “In this resource, a minimum size of 5kb was selected to limit the potential false positives viral classification of short sequences (23).”*

- Line 202: Most predicted taxa are Caudoviricetes. Can this be due to a reference database bias in the prediction tools?

*This is a very important question. We do expect most of the DNA viral diversity in human gut to belong to the Caudoviricetes group [see https://doi.org/10.3389/fmicb.2023.963173]. However, there is absolutely a bias the viral mining tool in their detection ability for rarer viral groups as described in our tool benchmark (*[*https://doi.org/10.3389/fmicb.2023.1078760)*](https://doi.org/10.3389/fmicb.2023.1078760))*. We clarified this important point in the discussion: L295-298: “The large proportion of vOTUs belonging to the Caudoviricetes class highlight the current need in exploring lesser-studied viral group, that are currently biased against by most viral mining tool (23).”*

- Add a figure summarizing datasets contributing to AVrC. For example proportions of healthy/unhealthy and adult/infant samples.

Thank you for this suggestion. We added this information in the Figure 3A

- Consider performing an analysis of overlaps between the new infant datasets and existing gut viral catalogues.

*Thank you for this suggestion. This was indeed important information missing from this manuscript. We now added this analysis L203-205: “Only 67,081 vOTUs of the AVrC (15% of the 449,859 vOTUs) clustered at least one sequence from the previously published catalogue and a viral sequence from our infant microbiome mining effort.”*

- Not needed to be addressed, I'm interested in seeing how much overlap there are in vOTUs between those in AVrC (gut microbiome) and other biomes in IMG/VR.
*Thank you for this interesting point. We expect little overlap with other environments, although some ecosystems such as wastewater viromes should have a good overlap in terms of phages identified and these found in the human gut. We absolutely will consider evaluating this overlap for the next version of the catalogue.*

**Discussion**
- The workflow presented was able to identify significant more vOTUS than other databses described in the introduction. i.e. Gregory et al analyszed 2.7K metagenomes with only 33K species vOTUS. Tisza et al. had 6K metagenomes for 45K species vOTU. With the infant dataset, you identified around ~600K vOTUs from nearly 8K samples. Briefly comment on the key differences in methodology that led to this increase in vOTUs identified.

*Thank you for this comment. The difference in vOTU retrieved can be attributed to (1) the use of recently developed viral mining tools such as DeepVirFinder able to accurately classify shorter sequence reads, and (2) the use of different length thresholds. We clarified this point in the result section for the infant mining effort description: L176-179: “It is important to note that our screen used a particularly lenient threshold of viral quality control compared to previous viral mining efforts, in order to allow for a broader retrieval of the viral diversity as additional quality filters were used when merging these putative viral sequences in the AVrC”*

- Looking at Fig 2, >90% of your sequences were low-quality/not-determined. Then in Fig 3, ~50% of vOTUS are low-quality/not-determined. Briefly discuss the impact of using these low-quality sequences for downstream analysis.

*Thank you for this comment. Indeed, we decided to use a lower length and quality threshold for the infant mining effort. This allowed to have a better recall of the viral population, probably at the cost of a higher false positive rate. Upon merging the infant viral sequences with the other resources, we chose to only keep vOTU for which the representative sequence had a length above 5kb. The vOTU may cluster several sequences, with the representative sequence being the longest of the cluster. This approach allowed us to exclude lower quality vOTUs from the AVrC and explains the difference between the Figure 2 and Figure 3 quality composition. We clarified this in the result section as previously described: L176-179: “It is important to note that our screen used a particularly lenient threshold of viral quality control compared to previous viral mining efforts, in order to allow for a broader retrieval of the viral diversity as additional quality filters were used when merging these putative viral sequences in the AVrC”*

- Line 255: Quantify "high prevalence" or rephrase. Most databases show minor plasmid contamination (<2%), with GVD being the highest at ~10%.

*We agree with the reviewer and changed the sentence to the following: L279-281: “When assessing the sequence quality of these previously published studies, we observed the consistent presence of potential plasmid sequences in the datasets, in particular for the oldest resources”*

-Line 260: Briefly summarize what is suggested by the benchmark of these detection approaches, and how it's relevent to your case of most of viral sequences are unique to 1 catalogue?

*Thank you for this comment. We clarified what we meant as follows; L286-288: “This result is consistent with previous observations suggesting the large impact of the chosen viral mining tool and computational approaches on the viral community composition retrieved (10).”*

-Line 284: The comparison between AVrC and UHGV needs to be expanded on. For example, the underlying datasets are very similar between UHGV and AVrC. See: https://github.com/snayfach/UHGV#data-sources. Highlight your strength and provide examples in terms of modularity, comprehensiveness, and usability that UHGV lacks.

*Thank you for this important point. We clarified the difference between the two resources as follows: L325-338: “Importantly, at the time of writing, other viral catalogue unification efforts are underway, in particular the Unified Human gut Virome Catalog (UHGV, available at* [*https://github.com/snayfach/UHGV*](https://github.com/snayfach/UHGV)*) that draw from similar data sources than the AVrC. However, the structure and aims of the AVrC and UHGC are distinct, and we believe the two resources to be complementary. Indeed, the UHGV focuses on providing a curated, high-quality reference dataset through stringent quality filtering and state of the art annotation pipelines and currently includes 168,570 vOTUs. In contrast, AVrC is designed as a comprehensive aggregation resource that preserves access to varying quality levels of viral sequences, allowing researchers to apply their own quality thresholds as needed. AVrC's modular architecture enables users to select sequence subsets based on multiple quality metrics, access both complete and partial viral genomes and apply custom filtering criteria through a dedicated command-line interface. The aim of the AVrc is to more easily integrate new viral sequences as they become available, as we believe that this flexibility is particularly valuable for research questions where non-complete viral sequences or lower-quality predictions might still provide valuable insights. Importantly, both approaches are addressing a current need to harmonize large scale viral catalogues into a consistent dataset.”*

-Touch on some ecological implications of what you noted in the gut virome, such as the dominance of Caudoviricetes and Spirochaetota-infecting phages, and what this may reveal about gut virome-host dynamics.

*Thank you for this comment, we now expended the discussion as follows: L295-304; “The large proportion of vOTUs belonging to the Caudoviricetes class reflects their central role in gut microbiome dynamics, particularly through their interactions with key bacterial phyla like Bacteroidota and Bacillota. Importantly, this also highlights the current need in exploring lesser-studied viral group, that are currently biased against by most viral mining tool (23). The identification of 393 vOTUs targeting Spirochaetota, a bacterial taxon found in non-industrialized populations, suggests that gut viromes may adapt to distinct microbial communities shaped by lifestyle and diet, and highlights the current gap in understanding of gut viromes from non-westernized population. The high proportion of predicted temperate vOTU in the AVrC confirms the importance of lysogeny in the human gut environment and confirms the need to also capture the prophage diversity associated to the human gut (30,31).”*

- Through out the discussion, there were mentions of bias in datasets/tools. How would you address those bias in future iteration of AVrC?

*Thank you for this comment, we now have added at the end of the discussion the following points: L339-349: ‘The overview of previously published mining efforts highlighted several gaps and biases in our current understanding of the human gut virome. First, the geographical bias towards adult western populations can be addressed by actively incorporating mining from underrepresented age groups and regions. Second, the impact of the computational viral detection tools is mitigated in the AVrC as the resource combines and allows to compare multiple detection methods, reducing tool-specific biases. The modular nature of AVrC will facilitates building upon these improvements, as new datasets and new detection or viral annotation methods can be integrated in the resource while maintaining backwards compatibility.’*

**Figures**
- Revise color themes for clarity, use distinct palettes for different plots within the same figure. Ensure semantic consistency between labels and colors. For example, in Fig 1A. Quality and Plasmid should using different color palettes. A 'complete' color in quality have a 'FALSE' label in plasmid, which conflicts on a semantic level.

*Thank you for this good point, we now updated the color scheme to avoid using the same color palette in the same plot in the Figure 1, 2 and 3.*

**Methods**
- Lines 304-305: Touch upon why were different assemblers used? Assembler choices does have an impact on the OTU prediction.

*Thank you for this very important comment. Different assemblers were used due to project-specific requirements and computational constraints. The large PRJEB70237 dataset (2328 samples) was assembled using Megahit v1.2.9, chosen for its memory-efficient handling of large datasets while maintaining competitive assembly quality. The remaining projects were processed through the METAnnotatorX2 pipeline using Spades v3.15, which can produce more contiguous assemblies but requires more computational resources. The use of different assemblers reflects a broader characteristic of the AVrC - its role as an aggregator of viral sequences obtained through diverse methodological approaches. While assembler choice can impact contig reconstruction and consequently vOTU prediction, the fundamental purpose of AVrC is to create a comprehensive resource that brings together viral sequences regardless of their computational origin. This is exemplified by our integration of multiple existing catalogues, each created using different assembly and viral detection pipelines. Our approach of clustering sequences at 95% identity over 75% of the shortest sequence helps create a unified resource while preserving the richness of viral diversity captured by different methodological approaches.*

- Include a workflow diagram summarizing steps for viral sequence identification, clustering, and annotation.

*Thank you for this comment. An overview of the computational workflow was now added in supplementary Figure 1.*

Supplementary Materials

- Supplement File 1: Some database references (e.g., MGV) are missing.

*We thank the reviewer for catching this issue. In the supplemental file 1, the references provided refers to the original cohort study, not the catalogue that mined that dataset. For some studies, this information could not be retrieved despite our efforts in manual curation. We now clarified the meaning of this column in the supplemental file 1.*

Web Interface
- The following could be due to shinyapps.io hosting issue for large datasets. Consider finding an alternative host that's more reliable:
* Download button on the side bar does not function.
* The filtering doesn't work, unchecking boxes then pressing search doesn't change the table.
- Align bullet margins under the "About/Viral Metadata Description" section.
- Use more human-friendly column headers in the table display.
- Consider adding a GIF/video tutorial for using the website.

*Thank you for these very good suggestions. We agree with the reviewer that the documentation was too light and that the use of a Shiny App is limited by the server memory capacity to interact with such a large dataset on the fly. Considering these points, we decided to re-organize the way we make the AVrC accessible to users:*

*- The database is directly accessible in Zenodo, along with a description of its content and associated data*

*- To help with the accessibility of the AVrC we developed a python CLI toolkit that allows users to download and subset the dataset. This replaces the Shiny App we had developed before. Indeed, as the reviewer noted, the app was slow and limited by the web implementation. The same functionalities are maintained through the CLI but are now more reactive and able to scale up to the dataset size. The CLI tool is available at https://github.com/aponsero/AVrC_toolkit*

*- The documentation for both the database and the CLI along with a tutorial on how to use the CLI have now been added in the Wiki section of the Github repository (*[*https://github.com/aponsero/AVrC_toolkit/wiki*](https://github.com/aponsero/AVrC_toolkit/wiki)*).*

Analysis code:
- Consider releasing the analyses code used throughout the manuscript.

*Thank you for this suggestion. We now have added the figure codes in a github repository along with the relevant data tables to reproduce the figures of this manuscript:* [*https://github.com/aponsero/Figures_AVrC_manuscript*](https://github.com/aponsero/Figures_AVrC_manuscript)

*The github repository link is now indicated in the data availability section of the manuscript.*
